# Robot-aided developmental assessment of wrist proprioception in children

**DOI:** 10.1186/s12984-016-0215-9

**Published:** 2017-01-09

**Authors:** Francesca Marini, Valentina Squeri, Pietro Morasso, Claudio Campus, Jürgen Konczak, Lorenzo Masia

**Affiliations:** 1 Motor Learning and Robotic Rehabilitation Laboratory, Department of Robotics, Brain and Cognitive Sciences, Istituto Italiano di Tecnologia, Via Morego 30, Genova, 16163 Italy; 2 Human Sensorimotor Control Laboratory, School of Kinesiology and Center for Clinical Movement Science, University of Minnesota, USA, 1900 University Ave S E, Minneapolis, 24105 USA; 3 School of Mechanical & Aerospace Engineering, Nanyang Technological University, Singapore, Singapore 639798

**Keywords:** Proprioception, Developmental changes, Robot-aided assessment, Joint position matching, Children, Wrist joint

## Abstract

**Background:**

Several neurodevelopmental disorders and brain injuries in children have been associated with proprioceptive dysfunction that will negatively affect their movement. Unfortunately, there is lack of reliable and objective clinical examination protocols and our current knowledge of how proprioception evolves in typically developing children is still sparse.

**Methods:**

Using a robotic exoskeleton, we investigated proprioceptive acuity of the wrist in a group of 49 typically developing healthy children (8–15 years), and a group of 40 young adults. Without vision participants performed an ipsilateral wrist joint position matching task that required them to reproduce (match) a previously experienced target position. All three joint degrees-of-freedom of the wrist/hand complex were assessed. Accuracy and precision were evaluated as a measure of proprioceptive acuity. The cross-sectional data indicating the time course of development of acuity were then fitted by four models in order to determine which function best describes developmental changes in proprioception across age.

**Results:**

First, the robot-aided assessment proved to be an easy to administer method for objectively measuring proprioceptive acuity in both children and adult populations. Second, proprioceptive acuity continued to develop throughout middle childhood and early adolescence, improving by more than 50% with respect to the youngest group. Adult levels of performance were reached approximately by the age of 12 years. An inverse-root function best described the development of proprioceptive acuity across the age groups. Third, wrist/forearm proprioception is anisotropic across the three DoFs with the Abduction/Adduction exhibiting a higher level of acuity than those of Flexion/extension and Pronation/Supination. This anisotropy did not change across development.

**Conclusions:**

Proprioceptive development for the wrist continues well into early adolescence. Our normative data obtained trough this novel robot-aided assessment method provide a basis against which proprioceptive function of pediatric population can be compared. This may aid the design of more effective sensorimotor intervention programs.

## Background

Proprioceptive signals from peripheral mechanoreceptors give rise to our awareness of limb position and movement in space [1]. Moreover, intact limb proprioception is essential for many aspects of motor control such as interlimb coordination [2, 3], for correcting and updating movement strategies [4] or for the formation of muscle synergies [5]. Proprioceptive signals are also crucial for motor learning [6, 7], providing information necessary for building and updating internal models of limb representation [8]. Proprioceptive loss or dysfunction caused by damage to the peripheral or central nervous system [9–12] in children has been shown to impair their motor control [13] and learning [7, 14]. A comprehensive knowledge of the typical development of human proprioception and its transition through adolescence into adulthood is currently lacking. However, given the importance that proprioception plays for motor development, it seems meaningful to characterize the course of its development, because such knowledge can be used as reference when diagnosing pediatric neurological disorders that are associated with somatosensory deficits [15]. Ultimately, the establishment of diagnostic standards and accepted functional assessment protocols would aid therapeutic intervention. Currently, an unbiased, accurate, and objective method to assess proprioception is still missing in clinical practice. Part of the problem arises from the fact that proprioceptive function is not easy to assess in children. The available tests either lack sensitivity, are difficult to understand, or they require a prolonged attention span that young children find difficult to maintain.

We here present a robot-aided method to assess joint position sense acuity for the three degrees of freedom of the wrist/hand complex in a cohort of typically developing children. The ability of such robotic device to provide valid and reliable proprioceptive measurements in adult subjects have been previously demonstrated [16]. The study seeks to document that the method is suitable to assess proprioceptive function in children and is capable to delineate developmental changes in limb position sense across childhood and adolescence. The task employed an ipsilateral joint position matching paradigm [17, 18] in which individuals must replicate a previously assumed reference joint position in the absence of vision, solely relying on proprioceptive information. Compared to traditional psychophysical methods that are typically time-consuming, a joint position matching task is particularly well suited for studying proprioceptive function in children [19], because task demands are low and the task itself is easy to understand. This test examines the acuity of position sense from two different aspects: accuracy and precision. With respect to a joint position matching task, accuracy indicates how close a sensed limb position corresponds to the true physical position of the wrist/hand complex and it is measured through the *matching error*. For a true response the *matching error* is equal to zero. In contrast, precision is represented with the *variability* which indicates the agreement between the repeated responses and the lower is the *variability*, the higher is the precision.

The aim of this study was threefold: (a) investigate if there are age related changes of proprioceptive acuity during development, (b) determine when children begin to reach adult levels of proprioceptive acuity, and lastly (c) characterize the isotropy/anisotropy of acuity across the three joint degrees of freedom of the wrist/hand complex (i.e. wrist flexion-extension, wrist abduction-adduction, hand supination-pronation).

## Methods

### Participants

Proprioceptive acuity was assessed in 49 healthy children (23 males, 26 females), from 8 years 1 month to 15 years 4 months and 40 healthy adults (mean age 28.9 ± 3.86 years, 21 females, 19 males). The participants presented no neuromuscular disorders and were naive to the task. The Edinburgh Handedness Inventory was completed to determine right hand dominance [20]. Experiments were carried out at the Motor Learning and Robotic Rehabilitation Lab of the Istituto Italiano di Tecnologia of Genoa, Italy and the study was approved by ethics committee of the regional health authority, Azienda Sanitaria Locale Genovese (ASL) N.3 (Protocol number 29/08 approved on 10/2/2008).

### Experimental setup

**Fig. 1:**
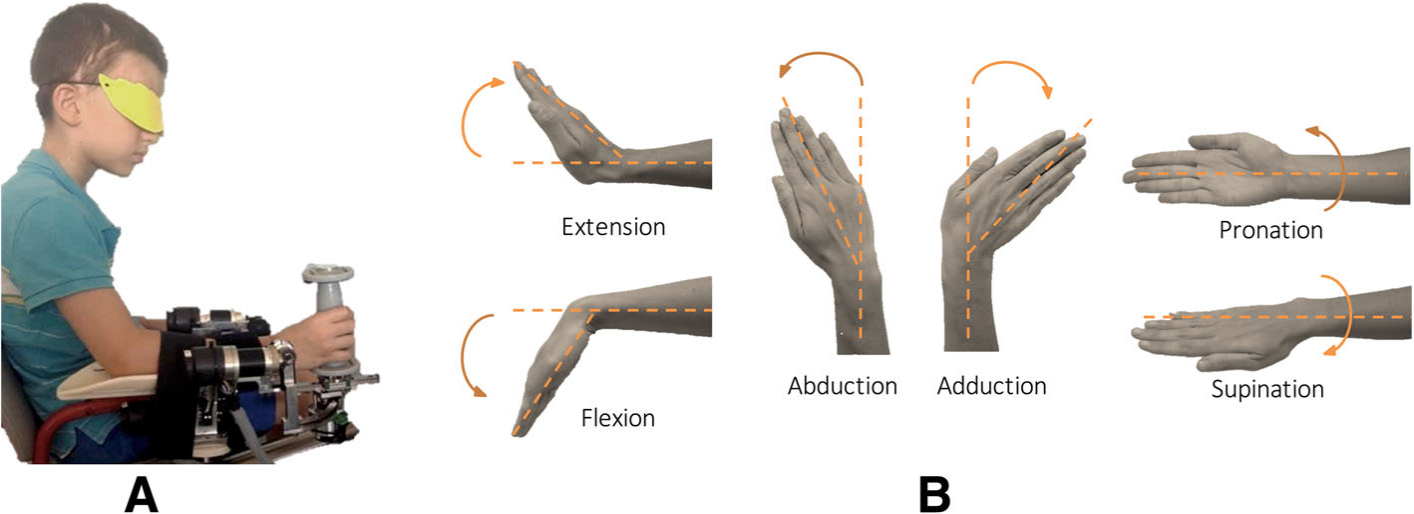
Wrist’s DoFs and participant. **a** Blindfolded participant performing the task. **b** wrist’s Degrees of Freedom tested

The experiments involved a behavioral task where subjects used a wrist haptic device [21], holding its handle with the right hand (Fig. 1 a); the robotic system allowed for movements along the three wrist articulations (Fig. 1 b), flexion/extension (FE), abduction/adduction (AA) and pronation/ supination (PS), for almost the full Range of Motion (RoM) of the human wrist: ±70° for FE, ±35° for AA and ±80° for PS. The robot is fully backdrivable, and it is powered by 4 brushless DC motors: two motors for abduction-adduction allowing for gravity compensation and one motor for each of the two remaining DoFs. An impedance control scheme was used to generate an assistive force field based on task requirements, with a 1 kHz sampling frequency for haptic rendering.

During the experiment subjects sat beside the robotic device with the frontal plane of their body aligned perpendicularly to the PS axis of the robotic device (Fig. 1 a). Position of the device was adjusted as to have a 120° angle between the upper and the forearm. Particular attention was given to the correct alignment between the axes of the robotic system and the anatomical joint axes of the wrist. The forearm was firmly strapped to a mechanical support to ensure repeatability of wrist positioning across the different trials and to avoid joint misalignment (slippage) during the experiment.

### Task and procedure

**Fig. 2:**
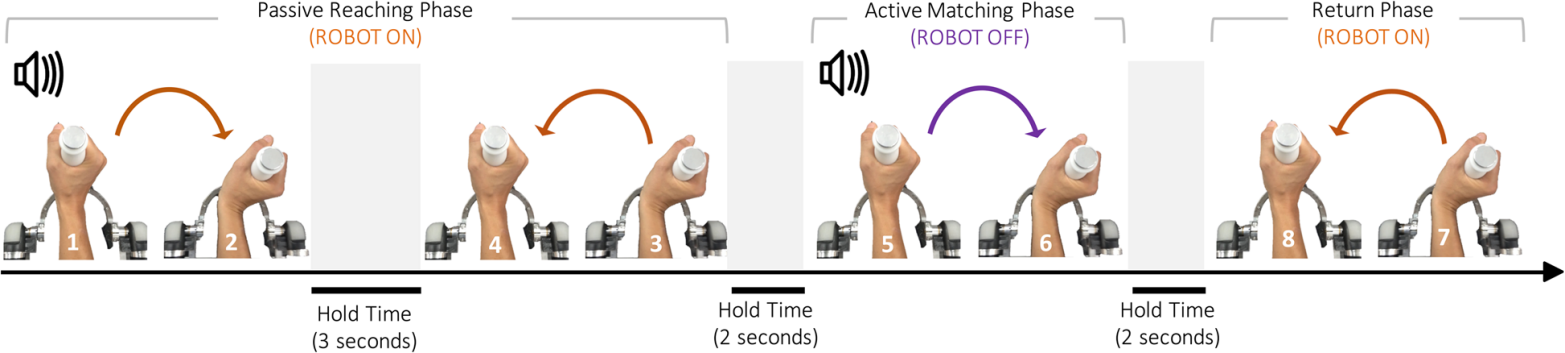
The Joint position matching task. Breakdown of each trial consisting in the passive reaching and active matching movements

Proprioceptive acuity was assessed with an ipsilateral Joint Position Matching (JPM) test [17], in this test, the wrist of a blindfolded participant was passively moved by the robotic device in a certain angular configuration. After a consistent holding time of three seconds [22, 23], the joint was passively returned to the initial start position and participants were then instructed to actively reproduce, as accurate as possible, the joint configuration previously experienced (Fig. 2). In this phase, besides the compensation of robot’s weight and inertia, no others forces or torques were applied to the wrist [24]. The active matching movement was considered completed when the end effector speed was below a 2°/s threshold for more than two seconds; then the robot moved the wrist back to the neutral joint angle and another trial was initiated with the same sequence previously described.

With the Joint Position Matching Task we tested the proprioception for all the three degrees of freedom: Flexion/Extension, Abduction/Adduction and Pronation/Supination (Fig. 1 b). Targets were located along the 6 anatomical directions corresponding to the 80% of the total functional wrist’s RoM. In particular, targets positions were: 32° for Flexion/Extension, 16° for Abduction/Adduction, 24° for pronation/supination. The targets across the three DoFs were presented in a pseudo-random fashion and each target was repeated 12 times for each DoF (12 for FE, 12 for AA and 12 for PS), for a total of 36 trials. Each trial started from the anatomical neutral wrist configuration (0°of FE, 0°of AA and 0°of PS). During active matching the robot allowed subjects to move freely only along the tested DoF while holding the other two in their neutral position. Auditory cues were used to indicate trial initiation and phases: a high frequency sound to mark the beginning of the 1st phase of the trial while a successive low frequency auditory feedback sounded indicating the subject that he/she could start moving and aiming to match the target location. As mentioned above, vision was occluded for all the duration of the experiment and subjects did not receive any feedback about their performance. To facilitate that attention to the task was maintained throughout testing, subjects were allowed to rest between 5 to 10 min after 12 trials. The overall experiment, including resting times, lasted between 40 to 60 min and was well tolerated even by the youngest children.

### Data analysis

Wrist joint rotations were recorded by the robot’s incremental encoders providing a resolution of micro radians. Acquired signals were post processed with a third-order Savitzky-Golay low-pass filter (cut-off frequency of 10 Hz) and converted to wrist angular displacement from the kinematics of the robot. To estimate wrist proprioceptive acuity and characterize the overall performance, two indicators were evaluated: the *Matching Error* [25] and *the Variability* [26].

The *Matching Error, ME* is the angular deviation from the proprioceptive target and it quantifies the performance accuracy during the active movement. It is defined as the absolute difference between the reference (proprioceptive target position) and the wrist matched angle:

1 $$  {ME} = \frac {{\sum\nolimits}_{i=1:N}|\theta_{i}-\theta_{T}|} {N}  $$

where *θ*_*i*_ is wrist’s final position of the *i*-trial, *θ*_*T*_ is the target position, averaged across the (*N*=12) repetitions of the same target (same DoF and condition).

The *Variability, V* measures the consistency, or precision, in terms of subject performance repeatability trial-by-trial during the active phase, and it is evaluated as the standard deviation of the wrist matched position (*θ*_*i*_) across all the (*N*=12) repetitions of the same target:

2 $$  {V} = StD(\theta_{i=1:N})  $$

To evaluate if subject’s position sense was influenced by learning, we performed linear regression analyses on ME with trial number as predictor variable.

3 $$  ME(trial_{number}) = a\cdot trial_{number}+b  $$

where *a* is the slope of the curve and *b* the intersect. If less than 25% of the variance in ME was explained by trial number and a slope close to zero was observed, then we concluded that no learning had occurred. We then performed three different analyses on ME and to answer the following questions.

*What is the best approximating function describing the developmental progression of proprioceptive acuity?* The first issue we sought to investigate was to characterize and mathematically express the way in which children proprioceptive acuity evolves with age. Therefore, in order to determine a function that accurately describes the developmental change, we performed multiple regression analyses of the matching error for each group of subjects, with the group age as the independent variable and identified the one that best fits the given set of data. In particular we compared four models:Linear:

4 $$ ME(age) = \,a\cdot age+b  $$

Exponential:

5 $$ f(age) = \,ae^{-age}+b  $$

Logarithmic:

6 $$ f(age) = \,a+b\log (age)  $$

Inverse-root:

7 $$ f(age) = \,a\frac{1}{\sqrt{age}}+b  $$

Goodness of fit was evaluated by computing the coefficient of variation (*R* ^2^), and the root mean square error (*rmse*) of the variance of the residuals.

*At what age does proprioceptive acuity reach the level of adults?* We were interested in establishing when the proprioceptive acuity of children plateaus at the adult level. We performed one-way ANOVAs with ME or V as response variables and age as factor. In case of significance, we performed pairwise comparisons of each age group against the adult group using Dunnett’s test to determine at what age the difference between the child age group and the adults was no longer significant.

*Is proprioceptive acuity isotropic or anisotropic across the three wrist DoFs? If yes, how the anisotropy is distributed?* To examine potential differences in proprioception among the three wrist DoFs, we computed the normalized measures of ME and V, according to the following equations:

8 $$ ME_{i\%} = \frac{ME_{i}}{\sum_{i=1}^{3} ME_{i}}  $$

For the *Matching Error* and:

9 $$ V_{i\%} = \frac{V_{i}}{\sum_{i=1}^{3} V_{i}}  $$

For the *Variability*, where *i* indicates the *i-th* DoF. The normalization allowed us to compare errors of different amplitudes across age groups.

## Results

**Fig. 3:**
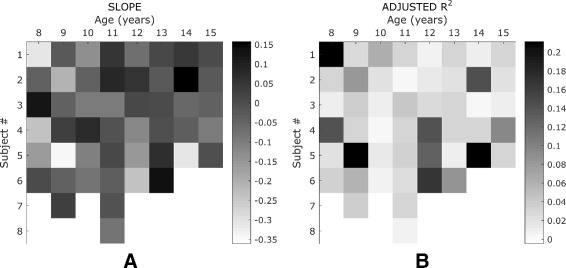
Analysis of learning trend. Slope of the fitting curve (panel **a**) and its goodness *R* ^2^ (panel **b**), for the *matching error* across the 36 trials for each subject. A decreasing slope of the linear fitting would indicate a positive learning rate, vice versa positive slope would point out a negative trend. The lower is the slope the flatter is the curve (the weaker is the evidence of a learning trend). Furthermore, low values of *R* ^2^ indicates that there is no a good fitting of the data, excluding the hypothesis of a learning trend

**Fig. 4:**
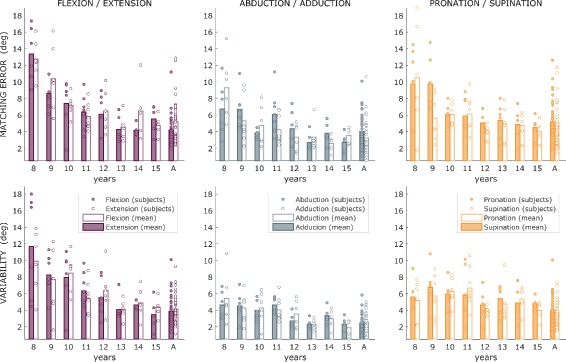
Analysis of raw data. Raw values for each participant (represented with *dots*) of *Matching Error* (*top panels*) and *Variability* for the three DoFs and their two directions (filled dots for one direction, empty dots for the opposite one). *Colored* or *empty bars* indicate mean value across subjects of *Matching Error* (*top panels*) and *Variability* in the two direction of the three DoFs

To determine if sensory learning had occurred during testing we investigated how ME changed over time. Regression analyses revealed that the respective slope *a* (see Eq. 3) for all the tested children never exceeded the boundary value of 0.3 (Fig. 3 a). Furthermore, the adjusted *R* ^2^ varied between 0 and 0.25 for all groups, confirming the absence of a consistent learning trend across trials (Fig. 3 b). Similar results were found for the adult group, which resulted to have no learning trend of *Matching Error*, neither in terms of slope of the fitting curve (*a*=0.0158±0.0107, mean and SE), nor in terms of adjusted *R* ^2^ (*R* ^2^=0.0381±0.00640, mean and SE). Overall results showed an increasing trend of performance from childhood into adolescence when proprioception matures, plateauing the adult level. The mean values of *Matching Error* and *Variability* are presented for each group and for the three wrist DoFs in Tables 1 and 2, respectively. Greatest errors were observed in the youngest children, with the mean values decreasing by more than 50% from 8 to 15 years of age for all the three DoFs. Figure 4 shows raw data of *Matching Error* and *Variability* for each subject and for the two opposite directions of the three wrist DoFs. No effect of motion direction within each DoF was found. Raw data show a highest variance of performance in the youngest groups. A one-way Analysis of Variance revealed a statistically significant main effect of age (*F*_8,240_=33.206 and *p*<0.001) among the nine groups (8 children groups and adults’ one).

**Table 1:** Mean and standard error for the *n* subjects of each age groups for Matching error

	Matching error		
	FE	AA	PS
8 ys (*n*=6)	13.13°±0.45°	8.09°±1.17°	10.20°±1.47°
9 ys (*n*=7)	9.57°±0.58°	6.04°±0.73°	7.62°±0.60°
10 ys (*n*=6)	7.48°±0.43°	4.35°±0.51°	6.08°±0.38°
11 ys (*n*=8)	6.06°±0.28°	5.16°±0.44°	5.99°±0.26°
12 ys (*n*=6)	6.29°±0.84°	3.84°±0.42°	4.59°±0.27°
13 ys (*n*=6)	4.41°±0.44°	2.91°±0.27°	5.15°±0.69°
14 ys (*n*=5)	5.32°±0.81°	3.17°±0.41°	4.83°±0.70°
15 ys (*n*=5)	5.10°±0.23°	3.09°±0.24°	4.26°±0.44°
Adults (*n*=40)	4.69°±0.25°	3.63°±0.25°	5.15°±0.32°

**Table 2:** Mean and standard error for the *n* subjects of each age groups for Variability

	Variability		
	FE	AA	PS
8 ys (*n*=6)	12.07°±1.75°	5.83°±0.78°	9.16°±1.5°
9 ys (*n*=7)	9.36°±1.08°	5.34°±0.58°	8.23°±0.60°
10 ys (*n*=6)	9.38°±0.50°	5.10°±0.60°	6.66°±0.62°
11 ys (*n*=8)	6.68°±0.37°	5.38°±0.23°	7.23°±0.38°
12 ys (*n*=6)	6.74°±0.85°	4.28°±0.43°	4.73°±0.43°
13 ys (*n*=6)	4.68°±0.32°	2.83°±0.18°	5.66°±0.62°
14 ys (*n*=5)	5.47°±0.42°	3.80°±0.53°	5.61°±0.72°
15 ys (*n*=5)	5.47°±0.42°	3.80°±0.53°	5.61°±0.72°
Adults (*n*=40)	4.41°±0.54°	3.21°±0.09°	4.76°±0.19°

**Fig. 5:**
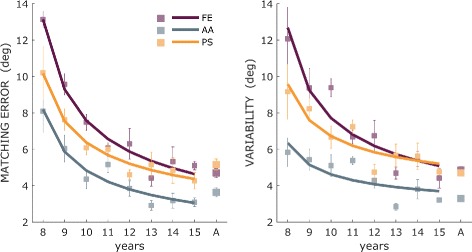
Developmental trend of *Matching Error* and *Variability*. Mean and standard error of *Matching Error* and *Variability* for each age groups and adults, for the three wrist DoFs. *Thick lines* indicate the inverse-root fit for these data across the age-range studied

*Curve-fitting analysis: shape of the developmental progression.* Such analysis indicated that development of sensorimotor-matching abilities with age is best approximated by the inverse-root model (see Eq. 7). Indeed, compared with all the other fittings curves of the *Matching Error* across ages, the inverse-root model provided the highest level of *R* ^2^ and the lowest value of *rmse* between age, as reported in Table 3. The inverse-root model provided an accurate fitting for the Variability as well (*R* ^2^=0.88 and *rmse*=0.91 for FE, *R* ^2^=0.57 and *rmse*=0.73 for AA, *R* ^2^=0.80 and *rmse*=0.72 for PS). The resulting curves of the inverse-root fitting for *Matching Error* and *Variability* (Table 4) are depicted in Fig. 5, for the three DoFs where is evident the improving trend in matching performance for increasing age.

**Table 3:** Goodness-of-fit statistics *R* ^2^ and *rmse* for the four different models: Eponential, Linear, Logaritmic and Inverse-root for the three wrist’s DoFs

	FE	AA	PS
Exponential	*R* ^2^=0.85	*R* ^2^=0.86	*R* ^2^=0.83
Fitting	*rmse*=1.12	*rmse*=0.66	*rmse*=0.80
Linear	*R* ^2^=0.72	*R* ^2^=0.77	*R* ^2^=0.74
Fitting	*rmse*=1.33	*rmse*=0.74	*rmse*=0.84
Logarithmic	*R* ^2^=0.93	*R* ^2^=0.91	*R* ^2^=0.93
Fitting	*rmse*=0.78	*rmse*=0.52	*rmse*=0.50
Inverse Root	*R* ^2^=0.96	*R* ^2^=0.92	*R* ^2^=0.96
Fitting	*rmse*=0.54	*rmse*=0.50	*rmse*=0.34

**Table 4:** Basic fit equation for Matching error and Variability (inverse-root model)

	$ f(age) = \,a\frac {1}{\sqrt {age}}+b $			
	Matching error		Variability	
	*a*	*b*	*a*	*b*
FE	13.5	–0.02	11.76	0.92
AA	7.96	0.23	4.09	2.24
PS	8.97	1.18	6.77	2.80

**Fig. 6:**
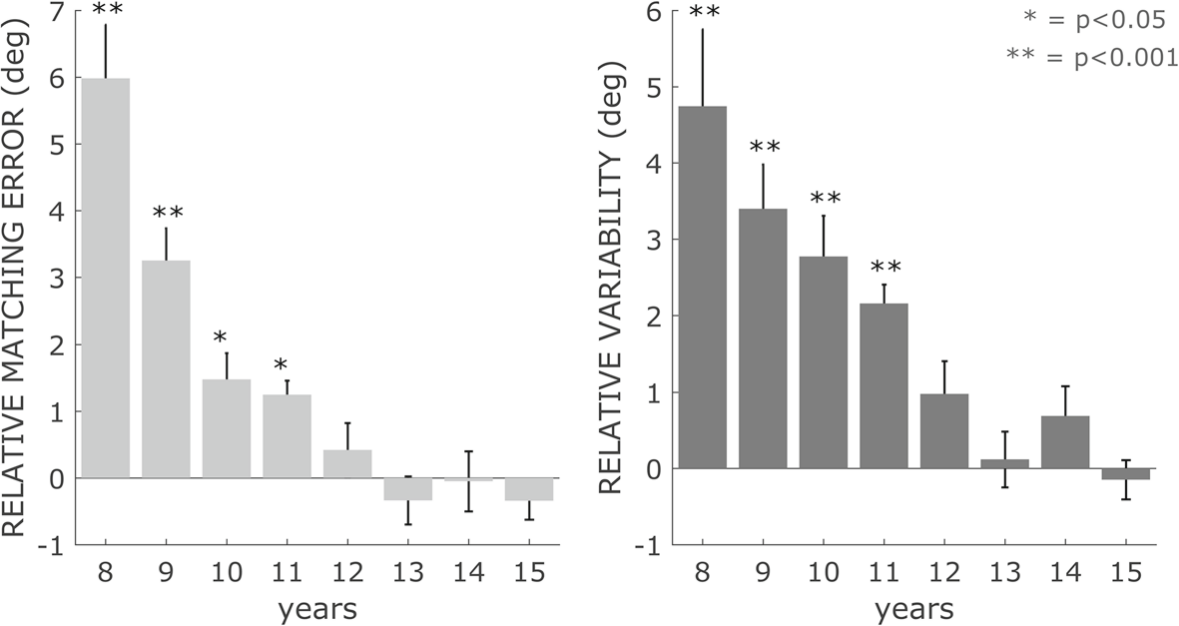
Relative *Matching Error* and *Variability*. Mean and standard error of the difference between *Matching Error* and *Variability* for each age group and the adults

*Transition to mature performance: plateau in proprioceptive performance.* Results indicated a change point at the age of 12 for both the *Matching Error* and *Variability*. Figure 6 reports the performance difference between each of the 8 groups of children and adults’ value of *Matching Error* and *Variability*: the bar plots graphs indicate that most of the improvement occurred between the ages of eight and eleven while older children presented a proprioceptive acuity comparable to that of adults. Dunnett’s tests among age groups demonstrated that children between eight and eleven years showed a performance, in terms of both *Matching Error* and *Variability*, that differs from those of adult with statistical significance, while the maturation of performance appears at twelve years of age, when comparison of the accuracy level results in no significant differences between children and adults.

**Fig. 7:**
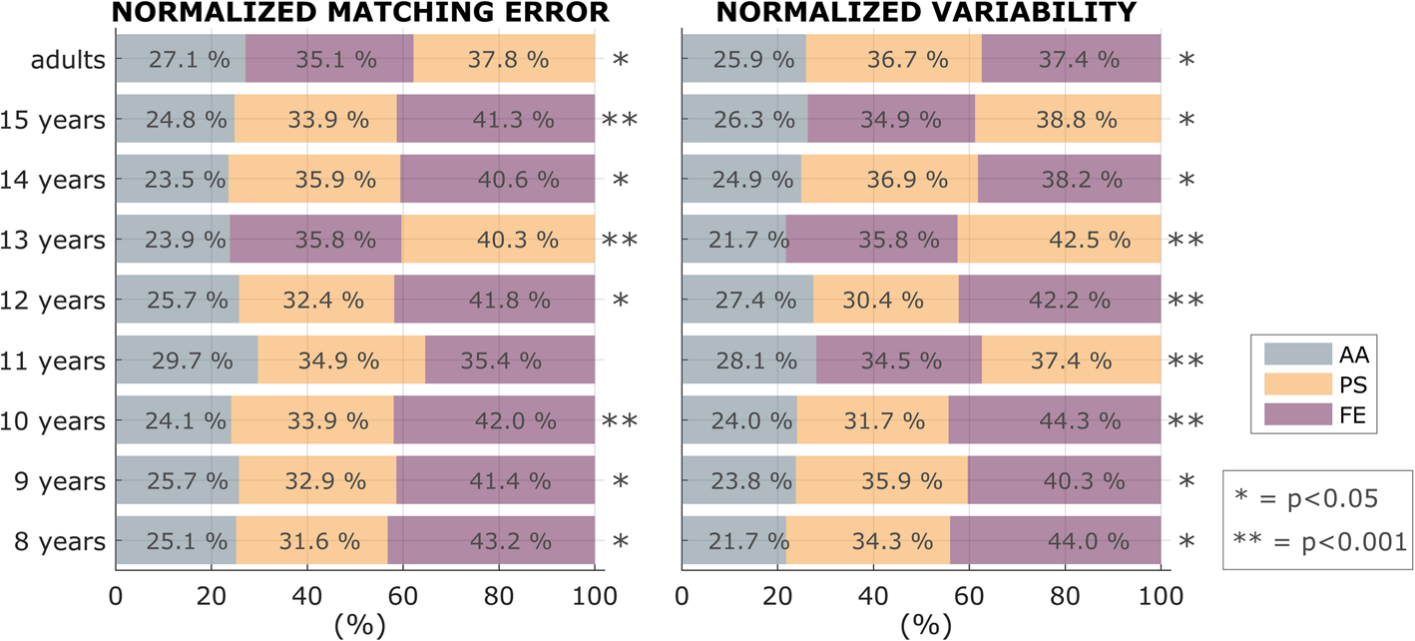
Anisotropy of wrist proprioception. Proprioceptive acuity of the three wrist DoFs. *Matching error* and *Variability*: AA is the most accurate DoF (smallest matching error) and the most precise (lowest Variability), compared with PS and FE. The contribution of each DoF is reported in ascending order

*Anisotropy of wrist proprioception.* Figure 7 clearly shows how proprioceptive acuity is anisotropic across the three DoFs. As for the adult group, the AA resulted to be the most accurate (lowest *Matching Error*) and the less variable (lowest *Variability*) for children at every age. No predominant trend resulted between FE and PS. This is confirmed by results of one-way ANOVA test, performed for each for each group to investigate the effects of the DoF. Results revealed statistical significance of the Normalized *Matching Error (* *ME*_*i*_ *%* *)*, for wrist proprioception anisotropy in all groups unless for groups eleven and fourteen years, while the Normalized *Variability (* *V*_*i*_ *%* *)* results to be significance among all the groups.

## Discussion

To more completely understand the nature of proprioceptive developmental changes across childhood and adolescence we measured proprioceptive acuity in 49 children and adolescents (8–15 age). Data collected from young adults served as reference to assess at what age adult levels of proprioceptive acuity were achieved. The main findings of the study are threefold: 1) The robot-aided task proved to be an easy and reliable method for measuring proprioceptive sensitivity in both children and adult populations. 2) Based on our empirical data we derived a mathematical model that best described the development of proprioceptive acuity from middle childhood to adolescence, and we identified that proprioceptive acuity continued to develop throughout middle childhood and early adolescence following an inverse-root law. Additionally, we found that this development plateaued and reached adult levels at approximately twelve years of age. 3) Wrist/forearm proprioception emerged to be anisotropic across the three DoFs with the abduction/Adduction exhibiting the highest levels of acuity.

The finding of a consistent refinement of wrist position sense with increasing age is in line with previous reports that focused on the assessment of age-related changes of proprioceptive acuity and sensorimotor ability [19, 27–29] and previous findings on cognitive development [30, 31]. Similar results have also been observed for precision of elbow position sense [32], which significantly decreases with age.

The age-related improvements in wrist position sense are related to several physical and physiological changes that occur during development. Proprioceptive percepts are based on the integration of signals from multiple peripheral receptors such as muscles spindles, tendon organs and or cutaneous receptors [33]. During development, these organs are subject to morphological changes [34] that alter their sensitivity and will influence the afferent information conveyed to the brain [35]. In particular, progressive improvements in proprioceptive acuity may reflect changes in central processes of sensory integration that are partially driven by experience-dependent consolidation of synaptic connections [28, 36], but may also reflect changes in the myelination of cortical fiber tracts [37, 38].

A further aspect to be considered is the gradual recalibration of the internal body schema in response to physiological and physical growth which may have an impact in proprioceptive matching accuracy of body in space [39]. It appears that updating internal models takes longer in children than in adults, or and that increasing experiences, increase one’s capacity for both speed and type of adaptation [40]. Indeed, it has been demonstrated that neural representations of limb dynamics in children are less precise than in adults (36), and internal models improves accuracy from birth to adulthood through an experiential learning [8, 24].

Finally, given that sensorimotor integration can be understood as a cognitive process, another interpretation for the improvement in the ability to integrate information from the proprioceptors can also be related to changes in cognitive functions from childhood through to adulthood, reflecting maturation of the central nervous system [41–43]. These improvements are supported by age-related developments in specific cognitive processes, most likely reflecting a refinement of the neural networks that support the most specialized aspects of cognition in adulthood [44] with a rate of development that varies across the different cognitive domains [45]. Previous studies found maturation of cognitive process from late childhood to adulthood to be best described by and exponential function [46] but, to our knowledge there are no researches providing mathematical models of development for proprioception. Our regression analyses demonstrated that the inverse-root descriptive function provided an optimal level of fitting accuracy between perceptual performance and age, yielding a better prediction than linear, exponential and logarithmic approximations.

At last another result emerging in our study concerns the anisotropy of wrist perception across its three DoFs. Outcomes showed the proprioceptive acuity of AA is significantly higher than the remaining DoFs (FE and PS). This anisotropy might be explained by differences in receptors, responsible of conveying sensory signals on joints’ movement [47, 48]. Indeed, immuno-histochemical studies of wrist anatomy revealed a high distribution density of mechanoreceptors in the dorso-radial ligaments such as dorsal radiocarpal, dorsal intercarpal, and scapholunate interosseous, a medium density in the volar and volar-triquetral, while the long radiolunate ligament is nearly void of mechanoreceptors [49]. The highly innervated dorso-radial ligaments are highly involved during AA, while the less innervated volar ligaments get primarily stressed during FE and PS. These differences in mechanoreceptor density and innervation might be responsible for the proprioceptive anisotropy reported in our investigation, and find confirmation in previous outcomes obtained by using the same robotic device [16, 50, 51].

Given that mechanoreceptors in ligaments are largely sensitive at the ends of the range of motion, rather than providing much information throughout the range of motion, one could claim that the improved performance for abduction\adduction is due to the lower distance of proprioceptive targets. However, even if the absolute location of the targets was different, their relative position, with respect to the amplitude of the range of motion, was the same for each DoF. Targets in the three DoFs were located at the 80% of the active RoM of each DoFs.

In conclusion, it could be argued that performance was influenced by fluctuation of subjects’ attention and by memory components. Attention and perception are certainly related process but this protocol, consisting in an active phase in which the subject has to move his/her wrist by him/herself, was developed also to keep the participant constantly focused on the task.

In conclusion, this study showed the feasibility of a robot-aided assessment of the limb position sense in children that yields objective data of proprioceptive acuity. The proposed robotic implementation of the JPM presents several advantages respect to the regularly administered proprioceptive assessment by a human therapist: a more reliable control on the stimulus, a higher accuracy and precise sensing of joints position and consequently an easier post processing of the results. The wrist robotic device allowed to deliver a rigorous and precise test, where each proprioceptive target associated to each stimulus was presented to the subject in a repetitive and accurate location consistently across repetitions. High resolution encoders, provide with the possibility to extract a reliable measure of performance and subjects’ proprioceptive acuity.

At present, there is no established objective method for clinical assessment of proprioception, and the few existing clinical rating scales such as the Nottingham Sensory Assessment [52] or the Rivermead Assessment of Somatosensory Performance [53] can provide only qualitative information and have low resolution. The method herewith proposed, proved to be reliable and easy to administer, and it could be introduced in clinical and rehabilitation practice as assessment tool to complement the afore mentioned clinical scales.

The absence of a learning trend through time indicate the test is robust and not influenced by fatigue or subjects’ familiarization with test and device.

## Conclusion

The robotic JPM test revealed that wrist proprioceptive acuity continues to change throughout childhood with a developing trend that follows and inverse-root law, and it reaches adult levels in early adolescence. While mapping the developmental time course of the wrist limb position sense contributes to our understanding of perceptual development, the methodology offers the opportunity to assess proprioceptive function in pediatric populations, which in turn may aid to improved therapeutic approach.

## Abbreviations

AA: Abduction/Adduction
DoF: Degree of freedom
EB: Error bias
FE: Flexion/Extension
JPM: Joint position matching
ME: Matching error
PS: Pronation/Supination
RoM: Range of motion
V: variability
